# The development of a questionnaire to assess the willingness of Chinese community health workers to implement advance care planning

**DOI:** 10.1186/s12904-022-01046-8

**Published:** 2022-09-09

**Authors:** Qunfang Miao, Bingyu Xing, Jingyi Li, Yanjuan Li

**Affiliations:** 1 School of Clinical Medicine, Hangzhou Normal University of Division of Health Science, The Affiliated Hospital of Hangzhou Normal University, No.126 of Wenzhou Rd, Gongshu District, Hangzhou, Zhejiang 310015 China; 2 School of Nursing, Hangzhou Normal University, Building No.8 in Shenyuan, No.2318 of Yuhangtang Rd, Yuhang District, Hangzhou, Zhejiang 311121 China; 3 Hangzhou Xianlin Vocational High School, No.12 of Xianfu Rd, Yuhang District, Hangzhou, Zhejiang 311122 China; 4 Hangzhou Hospital of Traditional Chinese Medicine, Hangzhou TCM Hospital Affiliated to Zhejiang Chinese Medical University, No.453 of Stadium Rd, Xihu District, Hangzhou, Zhejiang 310007 China

**Keywords:** Community health service centers, Medical and nursing personnel, Community health care workers, Advance care planning, Questionnaire, Reliability, Validity

## Abstract

**Background:**

To develop a questionnaire to evaluate the willingness of Chinese health care workers to implement an advance care planning (ACP) program for patients in a Chinese cultural context.

**Methods:**

Guided by the framework of the theory of planned behavior (TPB), a literature analysis and semi-structured interviews were conducted to create a pool of questionnaire items, and then the initial assessment questionnaire was developed by two rounds of expert consultations. A random sampling method was used to pre-survey 204 health care workers in community health service centers (CHSCs) in three urban areas of Hangzhou, Zhejiang Province. The final questionnaire was derived from item analysis and exploratory factor analysis.

**Results:**

Based on exploratory factor analysis, five common factors were identified from the questionnaire on community health care workers‘(CHWs) willingness to implement ACP. In general, the content validity of the questionnaire was 0.91, and the content validity of each of the entries ranged from 0.80 to 1.00, indicating acceptable overall questionnaire content validity. The total Cronbach coefficient for the questionnaire was 0.966, the Cronbach coefficient for each dimension ranged from 0.865 to 0.954, and the retest reliability was 0.856. The questionnaire produced a final draft containing five dimensions (behavioral attitudes, subjective norms, direct control, indirect control, and behavioral intentions) and 30 items.

**Conclusion:**

The questionnaire on the willingness of CHWs to implement ACP was validated and found to be reliable.

**Supplementary Information:**

The online version contains supplementary material available at 10.1186/s12904-022-01046-8.

## Background

Advance care planning (ACP) is one of the key elements of end-of-life care [1], with the basic notion of respecting patients’ medical autonomy, increasing quality of life [2]. Developed countries such as the United States and Europe have established appropriate policies and laws to ensure the implementation of ACP. The global concept of ACP is in constant development and palliative care units have been suggested as good locations for ACP implementation [3]. Primary health care facilities are suitable for promoting its implementation [4]. According to the Institute of Medicine, community health service centers (CHSCs) that provide patient-centered and family-oriented care are crucial from three perspectives [5]: sustainability, health care costs, and social outreach. These centers advocate a “whole community” approach to ACP development [5]. Furthermore, the community is an essential vehicle for implementing ACP.

The aging population in China has led to a growing demand for higher quality of death and palliative care services. Finkelstein released [6] the latest Global Expert Assessment of Quality of Death at the end of 2021, in which 81 countries were represented. Mainland China moved up from 71st in 2015 to 53rd. Therefore, it is evident that the level of quality of death in mainland China has advanced considerably but still requires improvement compared to other countries. The aging, empty nesting, and increasing disability and dementia of China’s elderly population have made the community a vital place for elderly and chronically ill patients to receive medical care [7]. The “Health China Action (2019–2030)” declares that Chinese residents’ health should be managed throughout their lives, and the value of ACP in community health services should not be overlooked [8]. However, the development of ACP in mainland China is still in its infancy, and there is no applicable legislation. One of ACP’s most notable achievements is the “Choice and Dignity” website for public welfare, which is operated by a private organization [9]. The majority of the current studies on ACP have been conducted in hospitals, with few studies involving communities. Moreover, the content of the community-based studies is descriptive in nature [10]. Implementing ACP relies on the participation of community health care workers (CHWs) who are in close contact with community residents. Understanding the viewpoint of CHWs is particularly relevant as primary health care workers are directly involved in treating patients with chronic disease and the elderly, who are more vulnerable to disease progression, frailty and physical decline. Community health care workers generally hold more positive attitudes toward ACP as they care for chronic disease patients and older adults in the community [8, 11, 12]. In addition, the implementation of ACP in other countries is not limited to elderly patients, cancer patients, end-stage patients, etc., but is often equally applicable to younger and healthier people [13]. Therefore, it can also be argued that ACP implementation is important to initiate healthcare-related conversations earlier in the disease trajectories.

At present, foreign evaluation tools have been developed to assess the willingness of health care workers to implement ACP [14, 15]. However, foreign assessment tools are not applicable in the current cultural context of China due to the sensitive nature of the ACP concept, the inevitable discussion of death-related topics, and factors such as Chinese filial culture and the lack of domestic ACP-related laws [16, 17]. A few studies have been performed in China, such as the questionnaire on the attitude and related behavior of advanced medical care staff in the oncology department compiled by Jiang Xiaoying et al. [18]. In contrast, Wu Yanyan et al. developed a questionnaire on the perception of living wills by geriatric nurses [19]. Wang Liying et al. developed a questionnaire on the attitude and related behavior of ACP staff in oncology department [20]. However, the above survey mainly targeted a specific group of health care staff in general hospitals, with relatively restricted dimensions. There is currently no suitable survey instrument for CHWs.

The theory of planned behavior(TPB)is one of the most fundamental and influential theories in the field of human behavioral intention research [21], explaining and predicting both individual behavior and behavioral intention [22]. Researchers have progressively applied TPB to healthcare populations [23–25], providing a theoretical framework for studying individuals’ willingness to perform behaviors [26–28]. One of the foundations for effectively implementing ACP in China [29, 30] is the development of a screening tool to assess the willingness of CHWs to implement ACP. We sought to develop a new measurement tool that would provide insight into the willingness of CHWs to implement ACP and report on the results of the validation of the tool.

## Methods

**Fig. 1:**
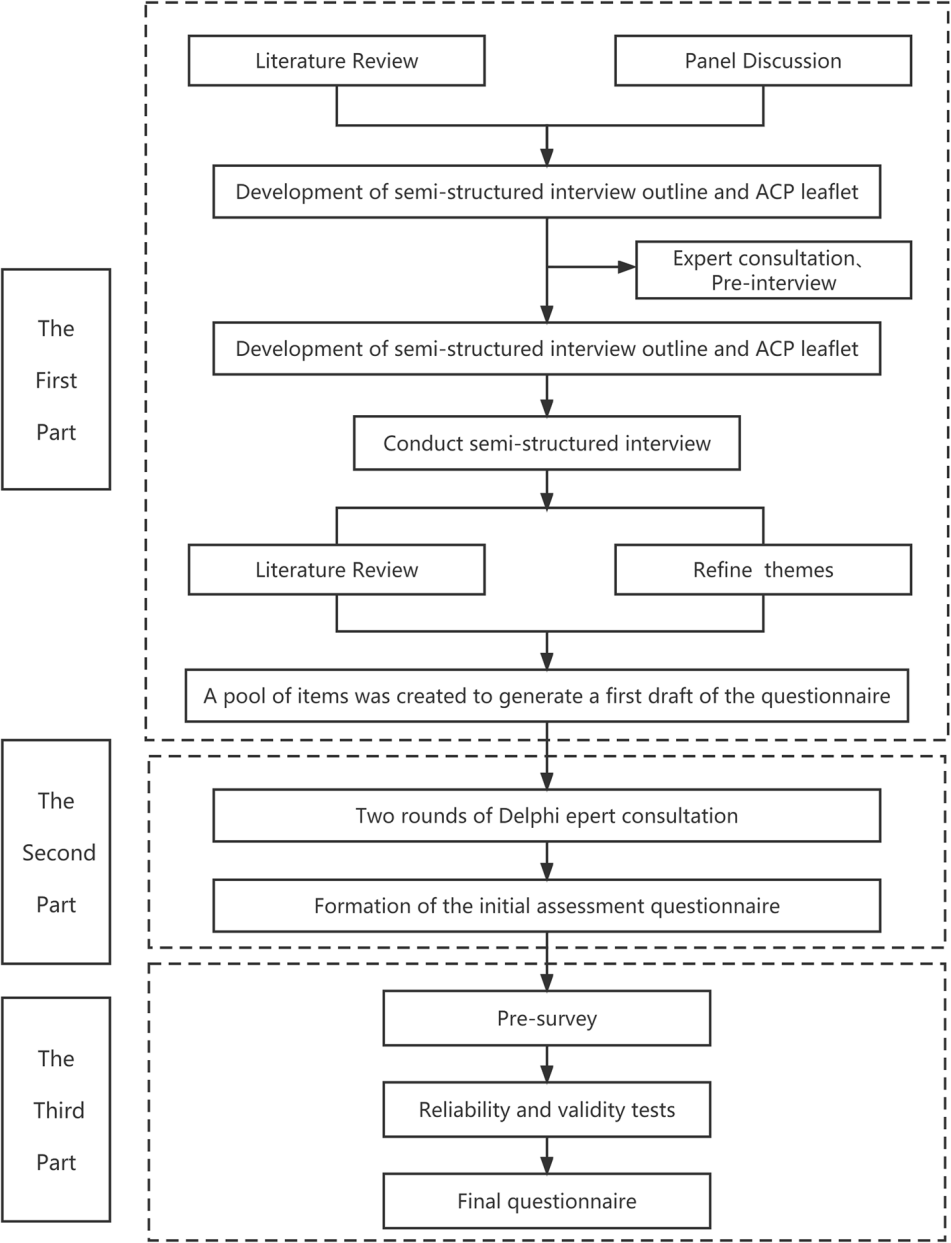
General flow chart of the study

The study was divided into three parts. The first part was to create the pool of items and generate the first draft of the questionnaire. The second part consisted of expert consultations to form the initial assessment questionnaire. The third part was to validate the initial measurement questionnaire (see Fig. 1).

### Phase 1: A pool of items was created to generate a first draft of the questionnaire

The PubMed, Web of Science, Science Direct, China Knowledge Network, Wanfang, and other database resources were searched for keywords such as “advance care planning”, “living will”, “advancement directive”, “implementation intention”, “influence factors”, etc. Furthermore, important policy documents on ACP, hospice, and end-of-life care that have been issued by the relevant government departments of various countries were reviewed. These documents were studied and used as the theoretical framework for our study. Based on the literature review and the Chinese cultural context, we developed an interview outline based on TPB and conducted semi-structured interviews with CHWs.

### Interview subject

A purposive sampling method was used to select health care workers from three community health service centers in Hangzhou. The inclusion criteria were community health service workers with one year or more of working experience who volunteered to participate in this study. The sample size was based on the principle of saturation of information [31].

### Data collection

The interviews were conducted in the office of the community health service center and lasted 30–40 min. In view of the novelty of the ACP concept, an ACP leaflet was distributed to the interviewees before the interview to help them understand the ACP concept and inform them of the purpose of the study. In the process of browsing the leaflet, the interviewer should avoid imposing his or her own understanding and judgment on the other person and provide explanation to the interviewee in neutral and objective language that is easy to understand. The information is organized and analyzed on the day after the interview.

### Analysis of interview results

**Fig. 2:**
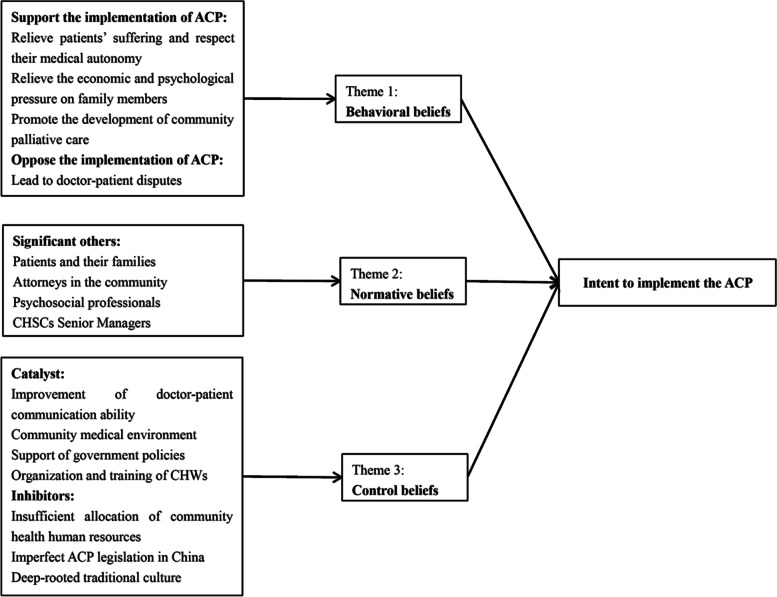
Results of interview themes and sub-themes based on TPB framework

By compiling and analyzing the interview data of 13 CHWs and combining the core concepts in TPB, the behavioral beliefs, normative beliefs, and control beliefs of CHWs about the implementation of ACP for patients were identified. These included 8 themes: positive evaluation of ACP, negative evaluation, support from patients themselves and their families, help from the community attorney team, assessment from psychiatric professionals, importance from community hospital leaders, facilitators, and hindrances (see Fig. 2).

### Phase 2: Expert consultation to determine the initial assessment questionnaire

In the Delphi expert consultation phase, the data collected from two rounds of expert consultation were organized and analyzed using the expert positivity factor, the expert authority level, and the degree of concentration and correspondence of expert opinions. According to the inclusion criteria of the experts, 10 experts representing 6 different fields, including geriatric care, community management, community nursing, clinical psychology and hospice, were selected to provide consultation in two rounds (see Table 1).

### Expert positive factor

In both rounds of expert consultations, 11 copies of the forms were distributed and recovered, with a 90.9% recovery rate in both rounds, which is well above 70% [32]. This indicates that the experts were very motivated to complete this study.

### Expert authority level

The expert authority coefficient is defined by the arithmetic mean of the expert’s basis of judgment and familiarity with the content. The expert authority coefficient in both rounds was greater than 0.80, which suggests a high authority.

### The degree of concentration of expert opinions

This metric is generally evaluated using the mean (Mj) and standard deviation (σ) of the importance scores of the items. In line with past studies [33], the screening criteria for questionnaire items were set to include responses that met both the coefficients of variation (CV) < 0.25 and Mj > 3.50. After the first round of correspondence, the Mj scores of the questionnaire dimensions ranged from 4.60 to 5.00, and the CV ranged from 0.00 to 0.11. According to the questionnaire modification criteria, the CV for items 8, 9, and 28 were 0.25, 0.25, and 0.38, respectively, and were removed. In the second round of correspondence, the importance scores of questionnaire dimensions showed Mj ranging from 4.20 to 4.90, with CV ranging from 0.07 to 0.22. The Mj of the importance scores of questionnaire entries of the second round of correspondence ranged from 3.70 to 5.00, with CV ranging from 0.00 to 0.26. In accordance with the questionnaire modification criteria, the CV for items 4 and 33 were 0.25 and 0.26, respectively, and were removed. Expert consultation was mainly conducted by email. After the first round of expert consultation, the experts’ opinions were summarized and collated. Statistical analysis of the ratings of the dimensions and entries filled out by the experts was performed, and the results were promptly discussed and communicated with the subject members to revise the entries in the questionnaire. Subsequently, the revised consultation form was sent to the experts for the second round of consultation. The experts were asked to rate the dimensions and entries of the questionnaire again and submit amendments in order to finalize each questionnaire dimension and entry.

### The degree of correspondence of expert opinions

The metric is predominantly expressed as a combination of CV and Kendall’s harmony coefficient (W). During both rounds of expert correspondence, the CV for each dimension were less than 0.20, and the experts’ opinions tended to be unified. The CV of items in both rounds of expert correspondence ranged from 0.00 to 0.38 and 0.00 to 0.26, indicating that some of the items in the questionnaire were agreed upon, while others were disputed. There was a positive correlation between the two rounds of correspondence between experts’ opinion dimensions with 0.269 and 0.305, and a negative correlation between the entries with 0.228 and 0.237, respectively, showing a statistically significant difference. This indicated that the experts’ evaluation of the questionnaire tended to be consistent and that the evaluation results were appropriate (see Additional file 1). After two rounds of expert consultation, a preliminary questionnaire was developed to measure the willingness of CHWs to implement ACP in four dimensions, covering the behavioral attitudes, subjective norms, perceived behavioral control, and behavioral intentions, with 33 items.

**Table 1:** Analysis of the importance rating of the first round of items by experts

Number	Items	Mj	σ	CV
1	I think the implementation of ACP can reduce the suffering of patients’ diseases.	4.80	0.42	0.09
2	I think the implementation of ACP will help respect patient medical autonomy.	4.90	0.32	0.07
3	I think the implementation of ACP can meet the wishes of the dying elderly.	4.90	0.32	0.07
4	I think the implementation of ACP can reduce the financial pressure on families.	4.40	0.52	0.12
5	I think the implementation of ACP can reduce the burden of decision-making for families.	4.70	0.67	0.14
6	I think the implementation of ACP can facilitate the development of community hospice care.	4.70	0.67	0.14
7	I think the implementation of ACP can save health care resources.	4.70	0.48	0.10
8	I think the implementation of ACP will increase my workload.	4.30	1.06	0.25
9	I think the implementation of ACP would add to my emotional burden.	4.20	1.03	0.25
10	I think the implementation of ACP will lead to disputes between doctors and patients.	4.50	0.06	0.01
11	The positive attitude of the patient towards ACP will motivate me to implement ACP.	4.80	0.63	0.13
12	The positive attitude of the patient’s family towards ACP will motivate me to implement ACP.	4.80	0.63	0.13
13	The help of a community attorney will motivate me to pursue ACP.	4.30	0.82	0.19
14	The support of my supervisors will motivate me to implement ACP.	4.80	0.42	0.09
15	The help of social workers (e.g. volunteers) will motivate me to implement ACP.	4.20	0.79	0.19
16	The help of a psychiatric professional will motivate me to implement ACP.	4.10	0.88	0.22
17	I have good communication skills to have ACP discussions with patients.	4.80	0.42	0.09
18	I have enough patience to support ACP discussions with patients.	4.60	0.70	0.15
19	I have enough responsibility to support ACP discussions with patients.	4.70	0.48	0.10
20	I have expert knowledge of psychological assessment to help me in my ACP discussions with patients.	4.50	0.53	0.12
21	I have enough ACP expertise to have an ACP discussion with my patients.	4.80	0.42	0.09
22	I am qualified to perform ACP.	4.60	0.70	0.15
23	At this stage, ACP has no formal regulations, workflow, relevant legislation, etc. in our country, which will hinder me in implementing ACP.	4.90	0.32	0.07
24	Inadequate community health human resource allocation will hinder my implementation of ACP.	4.70	0.48	0.10
25	Traditional culture (culture of death, culture of filial piety, etc.) will prevent me from implementing ACP.	4.70	0.67	0.14
26	Incentives for health care professionals will facilitate my implementation of ACP.	4.80	0.42	0.09
27	The support of national policy will facilitate my implementation of ACP.	5.00	0.00	0.00
28	The ethics of saving lives would prevent me from implementing ACP.	3.70	1.42	0.38
29	The family doctor contracting policy will facilitate my implementation of ACP.	4.20	0.92	0.22
30	A relaxed and comfortable community healthcare environment will facilitate my implementation of ACP.	4.70	0.67	0.14
31	Community hospitals conduct ACP organizational training sessions to promote my implementation of ACP.	4.60	0.70	0.15
32	A good doctor-patient relationship will facilitate my implementation of ACP.	4.50	0.85	0.19
33	In my future work, I would like to implement ACP in my community.	4.70	0.67	0.14

### Phase 3: Validation the initial test assessment questionnaire

To validate the previously developed questionnaire used to assess CHWs’ willingness to adopt ACP, a pre-survey was conducted.

### Research subject

- Overall study: CHWs in Hangzhou, Zhejiang Province, China.
- Inclusion criteria: People who have worked in community health services for one year or more and who are willing to participate in this study.
- Exclusion criteria: CHWs whowere absent from their posts.

### Research tool

General information questionnaire: There were 21 items in total, including age, gender, education, attitude toward death, and family physician services.

CHWs’ willingness to implement the ACP initial assessment questionnaire: The self-administered questionnaire included four dimensions, namely behavioral attitudes, subjective norms, perceived behavioral control, and behavioral willingness, which consisted of 33 items. Each item was scored from “strongly disagree” to “strongly agree” on a scale of one to five. Seven items were included in the behavioral attitude variables, of which six were positive and one was negative; the maximum score was 35 and the minimum was 7. A higher score indicates a more positive attitude toward ACP among CHWs and vice versa. There were nine subjective normative variables, all of which were positive, resulting in a maximum score of 45 and a minimum score of 9. Higher scores indicate that the support of significant others or groups or organizations has a greater impact on CHWs, and vice versa. Perceived behavioral control included 13 items, all of which were positive, with a maximum score of 65 and a minimum score of 13. Higher scores indicate that CHWs have more internal conditions and external resources to implement ACP, and vice versa. There were four positive entries assessing behavioral willingness; the maximum score was 20 and the minimum was 4. Higher scores indicate a greater behavioral willingness.

### Data collection

A random sampling method was used between June and August 2021 to randomly select three urban areas (Gongshu District, Shangcheng District, and Xiacheng District) from the five main urban areas of Hangzhou and a questionnaire survey was conducted among CHWs in these three urban areas. Due to the novelty of the ACP concept, informative leaflets were distributed to the subjects before the survey for better understanding, and the questionnaires were collected and checked carefully after completion. If any items were missing, the CHWs were consulted or corrected as soon as possible. Each questionnaire was carefully checked and two researchers collected and entered the data. Questionnaires that had more than 20% of items missing and unclear handwriting were considered invalid and were excluded.

## Results

### General information about the study subjects

A total of 210 questionnaires were distributed, and 204 were valid, with an effective recovery rate of 97.14%. According to the survey, the majority of respondents were female, comprising 82.8%; the majority of respondents were between the ages of 31 and 40, comprising 44.6% (see Table 2).

**Table 2:** General information of the study subjects (*n* = 204)

Characteristic		Number of people	N(%)
Sex	Male	35	17.2
	Female	169	82.8
Age	≤ 30	52	25.5
	31~40	91	44.6
	41~50	45	22.1
	> 50	16	7.8
Occupation	Doctor	91	44.6
	Nurse	113	55.4
Professional Title	Primary	100	49.0
	Middle	74	36.3
	Senior	30	14.7
Title	Yes	57	27.9
	No	147	72.1
Years of community work	1~5	59	28.9
	6~10	58	28.4
	11~20	56	27.5
	> 20	31	15.2
Education	Secondary School Degree	5	2.4
	College degree	34	16.7
	Bachelor’s Degree	161	78.9
	Master’s degree or above	4	2.0
Marital Status	Single	37	18.1
	Married	162	79.4
	Divorced/Separated	5	2.5
Child	No	54	26.5
	One	107	52.4
	Two or More	43	21.1
Religious beliefs	Yes	11	5.4
	No	193	94.6
Health Conditions	Healthy	125	61.3
	Occasional illness	76	37.2
	Frequent illness	3	1.5
	Chronic illness	0	0.0
	Disabled	0	0.0
Work experience in the general hospital or not	Yes	111	54.4
	No	93	45.6
Experienced bereavement or not	Yes	85	41.7
	No	119	58.3
Cared for the dying or not	Yes	57	27.9
	No	147	72.1
Heard of ACP or not	Yes	53	26.0
	No	151	74.0
Received ACP-related training or not	Yes	9	4.4
	No	195	95.6
Received Hospice-related training or not	Yes	43	21.1
	No	161	78.9
Received relevant training such as hospice or palliative care or not	Yes	27	13.2
	No	177	86.8
Death Attitude	Fear	36	17.6
	Acceptance	96	47.1
	Escape	72	35.3

### Program analysis

In this study, the critical ratio method was used and the results showed that the differences between the high and low groups for the other items were statistically significant (*P* < 0.05), except for the item 7 and the item 20.The results suggest the high quality of each item in the questionnaire overall. The Pearson correlation coefficient method test was used, and the results showed that the correlation coefficient of the other items were > 0.4 with *P* < 0.05, except for items 7, 20, and 29, which had correlation coefficients < 0.4. The above results indicate a good correlation between the items and the total score of the questionnaire, and the items demonstrated appropriate discrimination.

### Critical ratio method

The purpose of the critical ratio method is to calculate the critical ratio (CR) for the questionnaire entries [34]. 204 questionnaires were sorted from high to low scores; the first 27% were categorized as high scores (55 cases) and the last 27% as low scores (55 cases). Independent samples t-tests were performed and items 7 and 20 were removed as they demonstrated CR values < 3.0 or *P* > 0.05 (see Table 3).

**Table 3:** Critical ratio analysis of each item (*n* = 204)

Items	CR	*P*	Note	Items	CR	*P*	Note
1	17.197	0.000		18	16.019	0.000	
2	14.117	0.000		19	11.270	0.000	
3	14.483	0.000		20	2.419	0.017	Remove
4	15.105	0.000		21	14.720	0.000	
5	17.376	0.000		22	9.754	0.000	
6	15.547	0.000		23	12.425	0.000	
7	-1.694	0.093	Remove	24	10.602	0.000	
8	10.769	0.000		25	7.120	0.000	
9	12.020	0.000		26	12.004	0.000	
10	12.053	0.000		27	6.148	0.000	
11	15.270	0.000		28	6.703	0.000	
12	17.947	0.000		29	4.225	0.000	
13	18.762	0.000		30	10.898	0.000	
14	10.905	0.000		31	11.503	0.000	
15	19.039	0.000		32	11.762	0.000	
16	15.070	0.000		33	11.965	0.000	
17	13.461	0.000					

### Pearson correlation coefficient method

The Pearson correlation coefficient method was used to determine the correlation between each item and the total score of the questionnaire. If the correlation coefficient r is less than 0.4 or the correlation is not statistically significant, it indicates that the item is poorly discriminated and should be removed [34], so items 7, 20, and 29 are removed. After item analysis, a total of three items were deleted, and the final confirmed questionnaire items totaled 30 (see Table 4).

**Table 4:** Pearson correlation coefficients for each item

Items	r	Note	Items	r	Note
1	0.762**		18	0.793**	
2	0.773**		19	0.674**	
3	0.764**		20	0.196**	Remove
4	0.752**		21	0.733**	
5	0.755**		22	0.593**	
6	0.754**		23	0.691**	
7	-0.122	Remove	24	0.710**	
8	0.648**		25	0.556**	
9	0.677**		26	0.746**	
10	0.736**		27	0.479**	
11	0.771**		28	0.493**	
12	0.830**		29	0.372**	Remove
13	0.798**		30	0.674**	
14	0.703**		31	0.661**	
15	0.808**		32	0.700**	
16	0.774**		33	0.698**	
17	0.747**				

### Validity analysis

Exploratory factor analysis was performed on the questionnaire items. Five common factors were identified, with a cumulative variance contribution rate of 74.961%, and all 30 items showed a loading index > 0.4 [35]. I-CVI ranged from 0.80 to 1.00, and S-CVI was 0.91, indicating that the questionnaire on CHWs’ willingness to implement ACP had good content validity, and the entries accurately reflected the recipients’ opinions.

### Structural validity

**Fig. 3:**
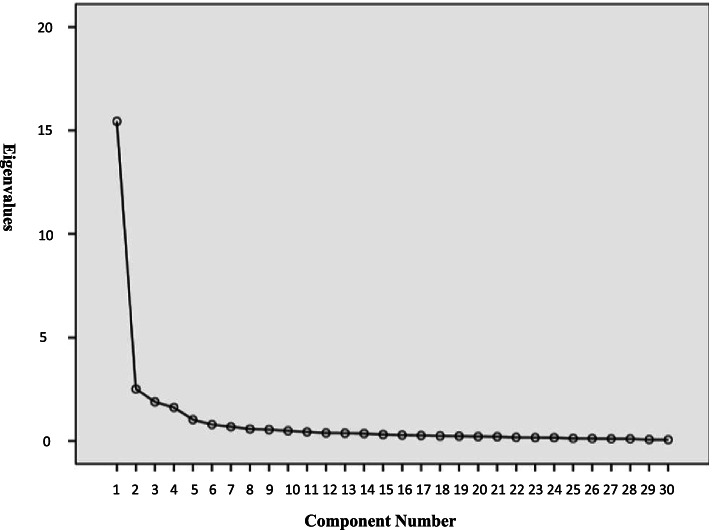
Factor analysis gravel diagram

The remaining 30 items were analyzed using exploratory factor analysis with a KMO of 0.936 and a c^2^ of 5901.161 as a result of Bartlett’s spherical test. The results demonstrated a statistically significant difference (*P* < 0.05), indicating that the scale was adequate for factor analysis (see Table 5). Using the rotated factor loadings (maximum variance method), taking eigenvalues greater than l.00 and the gravel plot, etc., a total of 5 common factors were extracted. A cumulative variance contribution rate of 74.961% was obtained, indicating the reliability of the factor model (see Table 6; Fig. 3; Table 7).

**Table 5:** KMO values and Bartlett’s spherical test results

KMO	Bartlett’s sphericity test for approximate chi-squared values	df	*P*
0.936	5901.161	435	0.000

**Table 6:** Total variance results

Dimensions	Initial Eigenvalues			Extraction of the sum of squares of loads			Sum of squared rotating loads		
	**Total**	**Variance percentage**	**Cumulative %**	**Total**	**Variance percentage**	**Cumulative %**	**Total**	**Variance percentage**	**Cumulative%**
1	15.440	51.466	51.466	15.440	51.466	51.466	6.335	21.117	21.117
2	2.513	8.378	59.844	2.513	8.378	59.844	5.119	17.062	38.179
3	1.888	6.293	66.137	1.888	6.293	66.137	3.955	13.182	51.361
4	1.619	5.396	71.533	1.619	5.396	71.533	3.767	12.557	63.918
5	1.028	3.428	74.961	1.028	3.428	74.961	3.313	11.043	74.961

**Table 7:** Factor loadings after rotation

Items	1	2	3	4	5
10	0.831				
11	0.792				
9	0.786				
12	0.757				
13	0.750				
14	0.733				
15	0.702				
16	0.642				
8	0.613				
3		0.840			
2		0.815			
4		0.796			
5		0.773			
6		0.741			
1		0.732			
27			0.797		
28			0.709		
26			0.706		
24			0.634		
25			0.609		
23			0.586		
19				0.774	
22				0.766	
21				0.725	
17				0.620	
18				0.586	
31					0.827
30					0.770
32					0.767
33					0.715

Based on the factor loadings of each item after pivoting, the number of factors and the factors to which each item belongs were generally consistent with the expected findings after factor analysis. Two common factors were identified in the “perceptual-behavioral control” dimension of the preset questionnaire as “direct control” and “indirect control”. Upon discussion of the results with the members of the group, the following final factors were identified:

- Public Factor 1: A six-item survey of CHWs’ attitudes toward the implementation of ACP by patients, entitled “behavioral attitudes”.
- Public Factor 2: A nine-item measure of the degree of influence of significant others or groups or organizations on the implementation of ACP by CHWs, entitled “subjective norms”.
- Public Factor 3: A five-item measure of the direct effect of internal self-efficacy on CHWs’ perceived behavioral control of ACP, entitled “direct control”.
- Public Factor 4: A six-item measure of the indirect effect of external environmental conditions on CHWs’ perceived behavioral control of ACP, entitled “indirect control”.
- Public Factor 5: A four-item measure of the willingness of CHWs to implement ACP with patients, entitled “behavioral intentions”.

### Content validity

A questionnaire I-CVI of 0.80 to 1.00 was used to assess the content validity of the remaining 30 items, exceeding the minimum standard of 0.78. In this study, the S-CVI was 0.91, which exceeded the well-defined criterion of 0.90 [36], indicating that the questionnaire content was valid.

### Reliability analysis

The Cronbach coefficient and retest reliability were used to evaluate the reliability of the assessment questionnaire. The Cronbach coefficient of the CHWs’ willingness to implement the ACP questionnaire was 0.966, and the Cronbach coefficient of the various dimensions ranged from 0.865 to 0.954. Since all the results were greater than 0.8, the questionnaire was internally consistent (see Table 8). The questionnaire was retested by thirty CHWs after a two-week interval, and the results demonstrated good reliability (0.856).

**Table 8:** Information on the Cronbach coefficient

Dimensions	Number of questions	Cronbach coefficient
1: Behavioral attitudes	6	0.954
2: Subjective norms	9	0.952
3: Direct control	5	0.904
4: Indirect control	6	0.865
5: Behavioral intentions	4	0.919
Total	30	0.966

## Discussion

Foreign research on community ACP is supported by legal, policy, and funding safeguards and support. The focus of the studies has shifted from conceptual relationship exploration, assessment tools, the current state of cognitive attitudes, and influencing factors to a series of targeted studies on the implementation of ACP interventions. In contrast, the development of ACP in China is still in the initial promotion stage, mainly based on the descriptive study of ACP. Chinese scholars He Ping et al. [37], Zhang Rong Rong et al. [11], and Yang Zhen et al. [12] conducted surveys on ACP cognition and attitudes of community residents, elderly people, and patients with chronic diseases. The results showed that community people have positive attitudes toward ACP and have the psychological basis to accept the concept of ACP. In this context, fewer studies have investigated the perceived attitudes and influencing factors of ACP from the perspective of CHWs. Wu Yuhua [38] conducted a survey and analysis of 120 nurses in a CHSC in Shanghai and found that community nurses had low knowledge of ACP. However, more than half of them had a favorable attitude toward ACP, and most supported its implementation and promotion as a policy. Zhang Dandan et al. [39] conducted a survey on CHWs’ knowledge, trust and behavior of ACP, and the results showed that CHWs had more positive attitudes toward ACP, but lower levels of cognition and behavior. The above study shows that different groups in the community have a positive attitude and some recognition of ACP, although their awareness is low.

In western countries such as the United States, social workers have mostly assumed the role of promoting, communicating, and assisting in the implementation of ACP and related documents to the community population [40]. However, the development of social workers in mainland China is not mature, and it is highly likely that the implementation of ACP will be taken up by CHWs. As the group with the most contact and communication with the community, CHWs are an important link between patients, families and the health care team, and thus can play a key role in distributing information about ACP and encouraging discussions among patients’ families about end-of-life care.

To answer the lack of ACP willingness assessment tools for community health care workers in China, this study developed a questionnaire for community health care workers to assess their willingness to implement ACP. This tool might help healthcare administrators and physician leaders identify the healthcare workers willing to implement ACP, and provide ACP training or join the ACP implementation team to maximize the positive effects of community healthcare workers in the community ACP model and further promote the development of ACP in our community healthcare services.

## Limitations

This study has several limitations. First, due to time, manpower and resource constraints, this study only surveyed health care workers in some community health service centers in Hangzhou, Zhejiang Province, and is not representative of the willingness of community health care workers to implement ACP nationwide.The willingness of ACP implementation may vary in different health care systems around the world. These various factors may have an impact on the generalization and applicability of the study results. In the follow-up study, not only should the questionnaire be culturally adapted, but also the sample area and sample size should be expanded so that the developed questionnaire to improve the applicability and generalizability. Second, the “Community health workers’ willingness to implement advance care planning questionnaire” is a Mandarin-validated questionnaire in English, and the English version has yet to be validated among English-speaking populations. Finally, ACP implementation is about advocating for and facilitating ACP. It remains to be seen whether those who score highly on the tool would, in practice, be more successful at promoting or facilitating ACP.

## Conclusion

The questionnaire developed to assess CHWs’ willingness to implement ACP shows good reliability and validity and can be used in survey research.

## Supplementary Information

**Additional file 1.**

**Additional file 2.**

## Data Availability

The dataset can be obtained on request from the first author, Qunfang Miao (854,623,272@qq.com).

## Abbreviations

ACP: Advance Care Planning
CHWs: Community Health Workers
TPB: Theory of Planned Behavior
CHSCs: Community Health Service Centers
CV: The coefficients of variation
CR: Critical ratio
