# Supplementary material for: The development of a questionnaire to assess the willingness of Chinese community health workers to implement advance care planning
Source: BMC Palliat Care. 2022 Sep 9;21:157. doi: 10.1186/s12904-022-01046-8 (PMC9461251; doi:10.1186/s12904-022-01046-8)
Supplement: Supplementary file 1 — Additional file 1. [file 12904_2022_1046_MOESM1_ESM.docx]

**Additional file 1：**Experts recommend adding items**（**DOCX 12kb**）**

**The first round of expert revision**

Under the behavioral-attitudinal dimension, **additional items** were suggested: 1 expert suggested adding the items "I believe that the implementation of ACP can maintain the dignity of the terminally ill or elderly", "I believe that the implementation of ACP can reduce the psychological stress of family members", "I believe that the implementation of ACP can help achieve the goal of hospice", and "I believe that the implementation of ACP can lead to family disputes". After discussion, the group concluded that "I think the implementation of ACP can maintain the dignity of dying patients or the elderly" and "I think the implementation of ACP can help achieve the goal of hospice" held similar meanings to entry 3, so this item was not added. The correlation between family disputes and the implementation of ACP by CHWs was not significant, so this item was not added. However, "I think the implementation of ACP can reduce the psychological stress of family members" was added.

Under the subjective normative dimension, **additional items** were suggested: 1 expert suggested adding the items "Help from hospice staff would motivate me to implement ACP", "Sharing from oncology caregivers would motivate me to implement ACP", and "Sharing from ICU staff would motivate me to implement ACP". After discussion, we decided to add the item "Help from hospice staff would motivate me to implement ACP". Considering that oncology caregivers and intensive care unit staff are not heavily involved in community health care services, and it is unlikely that they would be involved in ACP implementation，so the other two items were not added.，.

One expert suggested adding the influence of "groups and organizations" on the implementation of ACP by CHWs, such as the support of CHSCs or community councils and government administration and suggested adding "peer support". After discussion, the subjective norms should include groups or organizations in addition to the influence of important others on behavior. Therefore, the entries "The support of the community council will motivate me to implement ACP" and "The support of the government administration will motivate me to implement ACP" were added to the questionnaire. The ACP was created by a multidisciplinary team, and support from colleagues or peers may have a significant impact on the implementation of the ACP; so the item "Support from colleagues or peers would motivate me to implement ACP" was also included in the questionnaire.

**Additional items** were suggested under the behavioral intentions dimension. One expert suggested adding the items "In my future work, I would like to promote ACP to people in the community", "I would like to work on signing ACP documents", and "I would like to sign ACP documents myself ". After discussion, the group accepted the experts' opinions and added the items "In my future work, I would like to promote ACP to the community" and "In my future work, I would like to work on signing ACP documents". The purpose of the study was to explore the behavioral willingness of CHWs to implement ACP with patients and did not address the willingness of CHWs themselves to sign ACP, so the item was not added.

**The second round of expert revision**

**Add item**: 1 expert suggested adding the item "I am now willing to promote ACP to patients in the community". After discussion, the group accepted the suggestions of the above experts and changed the entries under this dimension to "I am willing to promote ACP to community patients now", "I am willing to start ACP discussions with community patients now", "I am willing to promote ACP to community patients in the future", and "I am willing to start ACP discussions with community patients in the future", respectively.
