# Supplementary material for: The development of a questionnaire to assess the willingness of Chinese community health workers to implement advance care planning
Source: BMC Palliat Care. 2022 Sep 9;21:157. doi: 10.1186/s12904-022-01046-8 (PMC9461251; doi:10.1186/s12904-022-01046-8)
Supplement: Supplementary file 2 — Additional file 2. [file 12904_2022_1046_MOESM2_ESM.docx]

**Additional file 2：**Community health workers' willingness to implement advance care planning questionnaire (DOCX 14kb)

**Community health workers' willingness to implement advance care planning questionnaire**

There is no right or wrong answer for this part of the question, where 1 is strongly disagree, 2 is relatively disagree, 3 is generally agree, 4 is relatively agree, and 5 is strongly agree.

**The following statements are about your attitude towards ACP, please tick the box of the option that matches your true opinion.**

1. I think the implementation of ACP can alleviate patients' disease suffering to some extent.
2. I think the implementation of ACP is conducive to respecting patients' medical autonomy.
3. I think the implementation of ACP will help to meet the wishes of patients with end-stage disease or elderly patients.
4. I think the implementation of ACP will help reduce the psychological stress of the families.
5. I think the implementation of ACP can facilitate the development of hospice care.
6. I think the implementation of ACP can reduce unhelpful treatment and overmedication.

**The following statements are about the extent to which people around you (e.g., community members, colleagues, leaders, etc.) or groups or organizations have influenced your implementation of ACP. Please check the box for the option that matches your honest opinion.**

1. The positive attitude of community residents towards ACP motivated me to implement ACP.
2. The help of community workers (e.g., volunteers, volunteers) will motivate me to implement ACP.
3. The help of psychological and related professionals will motivate me to implement ACP.
4. The help of the hospice staff will motivate me to implement ACP.
5. The support of community hospital leaders will motivate me to implement ACP.
6. The support of community hospital leaders will motivate me to implement ACP.
7. The support of the community council will motivate me to implement ACP.
8. The support from all levels of government administration will motivate me to implement ACP.
9. The media publicity about ACP will motivate me to implement ACP.

**The following statements are about whether you feel you are capable of implementing the ACP, please tick the box that matches your true opinion.**

1. I have a certain amount of patience to support my ACP discussions with patients.
2. I have a certain level of responsibility to support my ACP discussions with patients.
3. I have some psychological expertise to help me conduct ACP discussions with patients.
4. I have good communication skills to be able to have ACP discussions with patients.
5. I have the qualifications and work experience to be able to implement ACP.

**The following statements are about the degree of influence of external environmental factors on your implementation of ACP, please tick the box of the option that matches your true opinion.**

1. The support of national policy will facilitate my implementation of ACP.
2. Relevant departments organize ACP training sessions to promote my implementation of ACP.
3. Having the right environment for talks will facilitate my implementation of ACP.
4. A good doctor-patient relationship will facilitate my implementation of ACP.
5. Inadequate relevant laws and regulations will prevent me from implementing ACP.
6. Inadequate community health human resource allocation will hinder my implementation of ACP.

**The following statements are about your willingness to implement ACP, please tick the box that matches your true opinion.**

1. I am now willing to promote ACP to patients in the community.
2. I am now willing to open ACP discussions to community patients.
3. I am willing to promote ACP to my community patients in the future.
4. I am willing to start ACP discussions with community patients in the future.
